# Impact of virtual reality education on disease-specific knowledge and anxiety for hepatocellular carcinoma patient scheduled for liver resection: a randomized controlled study

**DOI:** 10.1097/JS9.0000000000001197

**Published:** 2024-02-21

**Authors:** Jaehun Yang, Jinsoo Rhu, Soyoung Lim, Danbee Kang, Heesuk Lee, Gyu-Seoung Choi, Jong Man Kim, Jae-Won Joh

**Affiliations:** a Department of Surgery; b Center for Clinical Epidemiology, Samsung Medical Center, Sungkyunkwan University School of Medicine, Seoul; c Department of Surgery, Gil Medical Center, Gachon University College of Medicine, Incheon; d VRAD Inc., Hanam, Korea

**Keywords:** hepatocellular carcinoma, patient education, virtual reality

## Abstract

**Purpose:**

Hepatocellular carcinoma (HCC) is a significant health concern, and the complexity of liver anatomy poses challenges in conveying radiologic findings and surgical plans to patients. This study aimed to evaluate the impact of a virtual reality (VR) education program on anxiety and knowledge in HCC patients undergoing hepatic resection.

**Method:**

From 1 January 2022 to 28 February 2023, 88 patients were enrolled in a randomized controlled trial, divided into the VR group (*n*=44) and the control group (*n*=44). The VR group received patient-specific 3D liver model education through a VR platform, while the control group underwent conventional explanation processes. Both groups completed preintervention and postintervention questionnaires assessing anxiety (using STAI-X-1, STAI-X-2, and VAS) and knowledge about liver resection. Comparison of the questionnaires were performed between the two groups. Multivariable logistic regression was performed to analyze factor related to decrease in anxiety.

**Result:**

While there was no significant difference in preintervention anxiety and knowledge scores between the two groups, the VR group exhibited significant reduction in STAI-X-1 scores (−4.14±7.5) compared to the control group (−0.84±5.7, *P*=0.023), as well as knowledge scores (17.20±2.6) compared to the control group (13.42±3.3, *P*<0.001). In the multivariable logistic regression model, VR education showed significant impact on decrease in STAI-X-1 score, postintervention. (OR=2.902, CI=1.097–7.674, *P*=0.032)

**Conclusion:**

The VR education program significantly improved knowledge and reduced anxiety among HCC patients compared to conventional methods. This study suggests that VR can be a valuable tool in patient education, enhancing comprehension and alleviating presurgical anxiety.

## Introduction

Highlights

- We investigated the impact of virtual reality as a platform for patient education.
- Patient-specific 3D visualization of the liver increased the patient’s knowledge.
- By the virtual reality program, anxiety level decreased significantly.

Hepatocellular carcinoma (HCC) is a major health problem with high incidence and mortality rates. Patients without cirrhosis who are diagnosed with HCC are usually treated with hepatic resection as the preferred treatment option^1^. Recently, surgery has shifted towards focusing on patient participation, rather than solely relying on the surgeon’s perspective^2^. Thus, communication between surgeons and patients is crucial for achieving optimal surgical outcomes^2^. However, studies have shown that patients often have difficulty understanding and retaining the information provided to them during consultations^2,3^. Especially, the complexity of anatomy of the liver makes it challenging to effectively communicate radiologic examination results (such as computed tomography and MRI) and surgical planning to patients.

Virtual reality (VR) technology has seen a significant increase in popularity in recent years, with a growing number of devices available for purchase and use by the public. While the entertainment industry has largely driven the expansion of VR technology, it has also shown promise in the medical field for a range of clinical applications^4^. VR-based education has been implemented in several studies within clinical practice, aiming to enhance patients’ comprehension of their medical condition and treatment procedures while effectively alleviating anxiety^5,6^. VR has the potential to improve comprehension of three-dimensional (3D) structures and establish an immersive environment that allows users to concentrate on the presented content.

Recent studies have demonstrated the efficacy of VR in enhancing clinical knowledge, patient understanding of procedures, and reducing anxiety in various clinical settings. An assessor-blinded prospective randomized clinical trial reported that VR-based preoperative education effectively reduced anxiety and information desire in patients awaiting elective surgeries, enhancing their overall satisfaction^7^. Another research showed the use of VR and 3D-printing in cardiac surgery patient education, which significantly reduced preoperative anxiety and improved patients’ understanding of their procedures^8^. A randomized clinical trial showed that VR interventions for adult patients undergoing elective surgery were effective in lowering preoperative anxiety and stress while increasing preparedness and satisfaction^9^. Demonstrated that VR interventions significantly reduced preoperative anxiety and postoperative pain in patients undergoing laparoscopic cholecystectomy. Participants exposed to VR, either through education or distraction, showed notable improvement in anxiety and pain management compared to those receiving routine care^10^. These findings demonstrate VR’s efficacy as a nonpharmacological adjunct in surgical patient care. However, the current evidence is restricted to specific surgical procedures and does not extend to establishing VR protocols for adult HCC patients undergoing liver resection.

Previous studies of VR education and patient knowledge relied solely on clinician reported outcome, no patient perspective outcome, and had limited before and after comparison, making it difficult to evaluate the effectiveness of VR education on knowledge and anxiety. Therefore, we conducted a randomized controlled trial to evaluate the effectiveness of VR education in improving knowledge and preventing anxiety among patients with HCC undergoing surgery.

## Methods

### Trial design and participants

We conducted an open label randomized controlled trial. Participants were patients with HCC scheduled to receive surgical resection between 1 January 2022 and 28 February 2023.

We excluded patients who had any of the following conditions: age equal or older than 70-years, and who previously underwent operation for HCC. The primary endpoint of this study is the improvement of surgical-related knowledge before and after education. We hypothesize that VR-based education will show a moderate effect size (Cohen’s D=0.5) compared to conventional education methods. To demonstrate this hypothesis with 90% power and an alpha of 0.05, 44 patients per group are required. Anticipating a dropout rate of 10% due to factors like the inability to undergo education, we aim to enroll 50 patients per group (total of 100 patients). The study was approved by the Institutional Review Board (IRB No. 2021-11-017-007), and all study participants provided written informed consent. The study protocol was registered at CRIS.nih.go.kr before the start of participant enrollment. Written informed consent was obtained from the patient for publication of this study. A copy of the written consent is available for review by the Editor-in-Chief of this journal on request. The study has been reported in line with Consolidated Standards of Reporting Trials (CONSORT) Guidelines^11^ (Supplemental Digital Content 1, http://links.lww.com/JS9/C3).

### Random allocation and blinding

A random allocation sequence was generated by a statistician not involved in patient recruitment using Sealed Envelope Ltd. 2019. Consenting patients were randomly assigned in a 1:1 ratio to VR education or usual clinical practice, using randomly permuted blocks of sizes 2 and 4.

An independent statistician transferred the randomization information into an Excel file and locked it. Study coordinators responsible for enrolling participants could not access the randomization codes and the locked information was not available until the patient was recruited. A total of three doctors participated for patient education. The doctors participated for both the VR group and the control group. Patients and investigators were not blinded to the nature of the intervention during the trial.

### VR education program

We used the Oculus quest 1 (Meta, Menlo park, CA, USA). as the investigational VR device. VRAD (Hanam, Korea) developed the VR platform which allowed multiuser-access. To generate the 3D liver model, we utilized Mimics Medical software (Materialise, Leuven, Belgium) and then imported it into the VR platform using Unreal Engine 4 software (Epic Games, Potomac, MD, USA).

**Figure 1:**
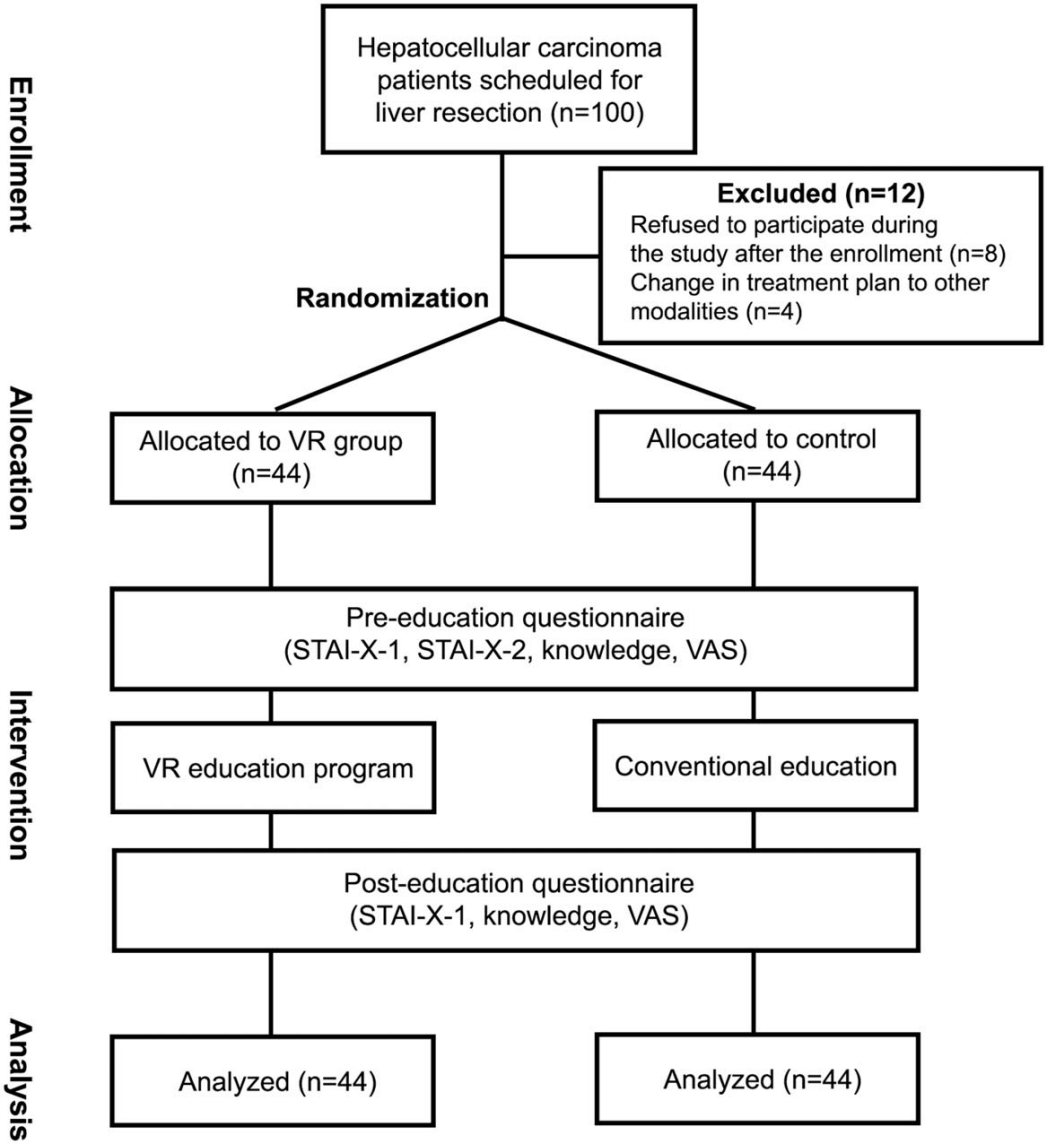
Flow diagram of the study. STAI-X-1, State-Trait Anxiety Inventory-X; VR, virtual reality; VAS, visual analog scale.

Within the VR environment, we designed an education room that closely resembles our hospital’s actual education room (Figure 1, Supplementary Video, Supplemental Digital Content 2, http://links.lww.com/JS9/C4). In the center of the virtual space, a 360-degree rotating model of the patient’s liver was shown. The doctor can adjust the transparency of the liver parenchyma, enabling patients to see internal structures such as the portal vein, the bile duct, the hepatic vein, and the tumor. The doctor explained the anatomical characteristics of the patient’s liver, the location of HCC, and the surgical plan while rotating the liver model 360 degrees in a 3D virtual space. Animations in the form of question and answer (Q&A) were created for the following six topics as follows: 1) What is the liver and why does HCC occur? 2) How is HCC treated with surgery? 3) Will the liver grow up after resecting it for the treatment? 4) What is the difference between open surgery and laparoscopic surgery for the treatment of HCC? 5) Does liver resection remove the gallbladder unconditionally? and 6) What are the possible complications after liver resection? An 8 min and 34 s video composed of six clips were played on the screen in the education room in the virtual space. The information included in the educational video were designed based on the interview of nursing staffs who mainly run the basic education for the patients undergoing liver resection. The facts that the patients were mostly confused as well as most frequently asked questions were asked to the staffs.

The doctor can control the educational program by interacting with the 3D model and playing the associated education videos. On the other hand, patients and their families can only passively watch and listen to the educational content provided within the VR platform.

### Control

Patients in the control group received the same clinical treatment as the intervention group except for VR education. The doctor who participated for VR group education also educated the patient before operation and gave information about the tumor location, surgical planning as well as the risk of complications after the operation. Overall, same information was provided to the control group compared to the VR group. Questions were allowed and answers were given. However, this information was given with the written information along with informed consent.

### Study outcomes

The primary outcome was knowledge evaluated before and after the education session. The change in the knowledge score after the education as well as the score itself was compared between the two groups. The knowledge questionnaire, specifically developed by our research team, was administered, encompassing inquiries pertaining to both general knowledge of liver resection and patient-specific information. The questionnaire consisted of 13 questions with the highest score being 20 (Supplementary Information, Supplemental Digital Content 3, http://links.lww.com/JS9/C5).

The secondary endpoint was anxiety before and after intervention. To evaluate anxiety levels, the Korean version of State-Trait Anxiety Inventory-X (STAI-X)-1^12^, STAI-X-2, and the visual analog scale (VAS, with a range of 0 for no anxiety to 10 for extreme anxiety), were employed.

### Statistical analysis

Comparison between the VR group and the control group was performed using appropriate statistical methods. The variables that were compared included knowledge score preintervention and postintervention, as well as mean change in knowledge score. Preintervention and postintervention score of STAI-X1 as well as the mean change of X-1 score were compared between the two groups. STAI-X2 was only compared before the intervention. VAS score preintervention and postintervention as well as mean change in VAS score was compared. Satisfaction score was not compared but only described in the VR group.

For further analysis, comparison between patients with or without decrease in STAI-X1 score was performed. Baseline characteristics as well as preintervention, postintervention, and change in score of questionnaires were compared between the groups. Satisfaction score was also compared between the two groups.

To analyze factors related to the decrease in anxiety after the education program, multivariable analysis using logistic regression was performed. Factors showing *P*-value less than 0.200 in the univariable analysis were included in the multivariable analysis. Backward likelihood ratio was used during the multivariable analysis.

For continuous variables, student’s *t*-test was performed. For categorical variables, *χ* ^2^ test and linear-by-linear association test was performed.

Two-tailed *P*-values less than 0.05 were considered statistically significant. The statistical analysis was performed using SPSS software version 25.0 (IBM).

## Result

### Patient and clinical characteristics

**Figure 2:**
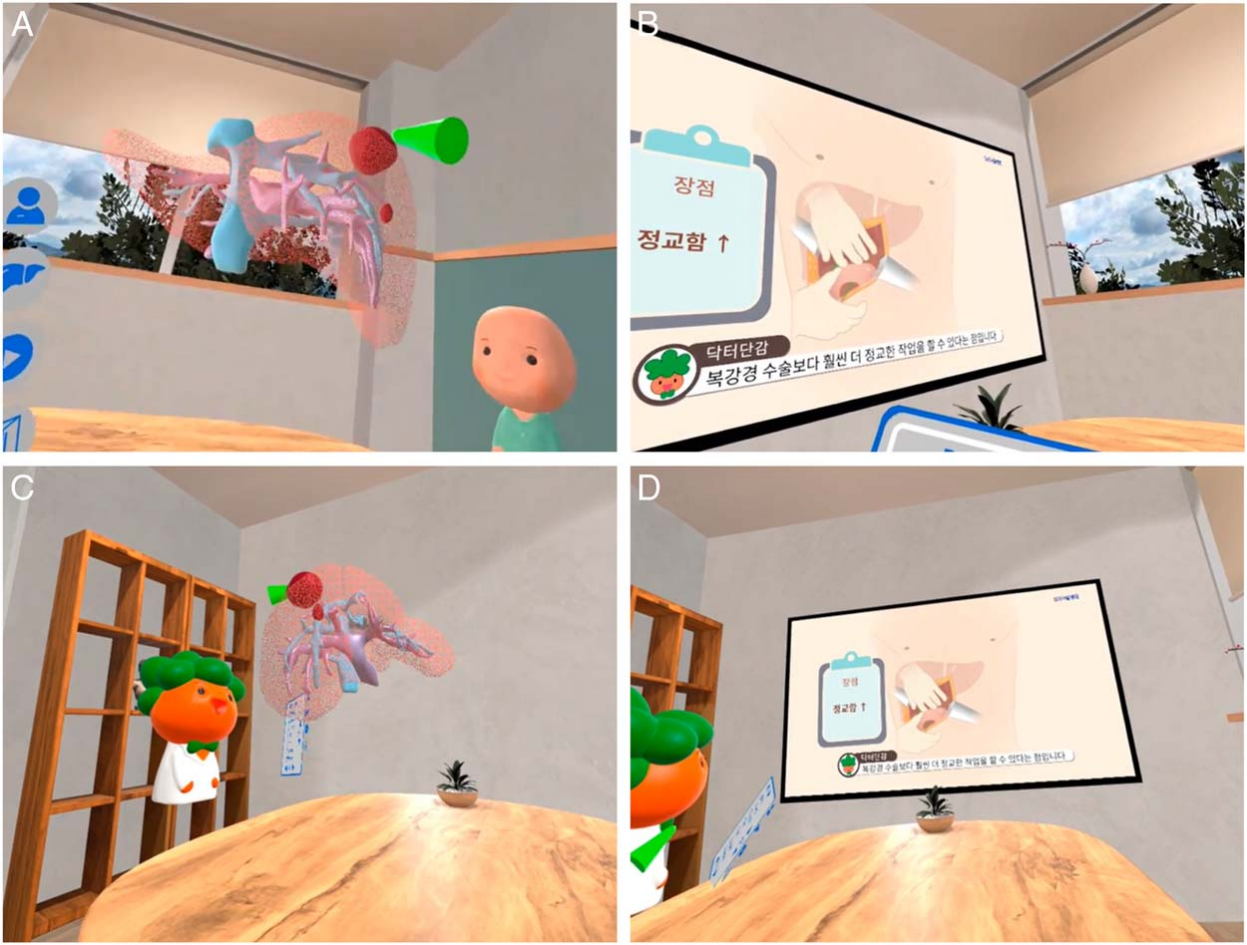
Virtual reality education room. (A) Doctor’s view while explaining the patient-specific 3D liver model. (B) Doctor’s view while watching the education video. (C) Patient's view while listening to the doctor’s explanation on patient-specific 3D liver model. (D) Patient’s view while watching the education video.

During study period, 100 HCC patients met the eligibility criteria and 100 (100.0%) agreed to participate and were randomly assigned to the VR group (*N*=50) or control (*N*=50) groups (Fig. 2). Before the initial intervention, four participants in the intervention group and four participants in the control group refused to undergo the intervention. The treatment plan for two patients in each group have been changed after the study enrollment. Therefore, a total of 12 patients were excluded from the study after screening. Consequently, a total of 88 patients, 44 in the VR group and 44 in the control group completed the study protocol (Fig. 2).

The mean age (±SD) of the 88 study participants (44 in the intervention group and 44 in the control group) at baseline was 58.1 (±7.7) years; 75.0% of participants were male. The mean ages of the VR group and the control groups were 57.5±8.0 and 59.7±7.3 years, respectively. The educational backgrounds were similar between the groups, showing same proportion of patients qualifying above college education. (both, *n*=23) Most of the patients had underlying liver disease of chronic hepatitis B (77.3%, *n*=34 in the VR group and 78.4%, *n*=35, in the control group). There were 15.9% (*n*=7) of patients with TACE, 11.4% (*n*=5) of patients with RFA, and 2.3% (*n*=1) of patient with RT performed before the operation in the VR group. On the other hand, 4.5% (*n*=2) of patients underwent TACE before the operation. Initially planned surgical extent was <10% in 18.2% (*n*=8) in the VR group, and 11.4% (*n*=5) in the control group. Initially planned surgical extent of 70% were 27.3% (*n*=12) in the VR group and 11.4% (*n*=5) in the control group (Table 1).

**Table 1:** Patient and clinical characteristics.

	VR group (*n*=44)	Control group (*n*=44)
Age (years)	57.5±8.0	59.7±7.3
Male	32 (72.7%)	34 (77.3%)
Educational background		
Elementary school	3 (6.8%)	1 (2.3%)
Middle school	3 (6.8%)	6 (13.6%)
High school	15 (34.1%)	14 (31.8%)
College	21 (47.7%)	22 (50.0%)
Graduate school	2 (4.5%)	1 (2.3%)
Previous psychiatric history	4 (9.1%)	1 (2.3%)
Etiology of HCC		
Hepatitis B virus	34 (77.3%)	35 (78.4%)
Hepatitis C virus	3 (6.8%)	2 (4.5%)
Alcohol	3 (6.8%)	2 (4.5%)
Non-B, non-C	3 (3.8%)	4 (9.1%)
Hepatitis B virus and alcohol	1 (2.3%)	1 (2.3%)
Previous treatment		
Transarterial chemoembolization	7 (15.9%)	2 (4.5%)
Radiofrequency ablation	5 (11.4%)	—
Radiotherapy	1 (2.3%)	—
Planned surgical extent		
<10%	8 (18.2%)	5 (11.4%)
15–20%	9 (20.5%)	25 (56.8%)
33%	13 (29.5%)	7 (15.9%)
50%	2 (4.5%)	2 (4.5%)
70%	12 (27.3%)	5 (11.4%)

HCC, hepatocellular carcinoma; VR, virtual reality.

**Figure 3:**
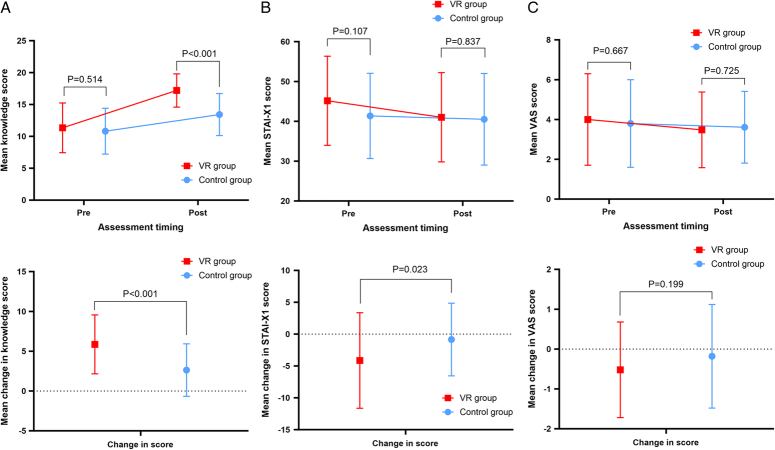
Comparison of mean values between two groups. (A) Pre-education and posteducation mean knowledge score between the VR group and the control group. (B) Pre-education and posteducation mean STAI-X1 score between the VR group and the control group. (C) Pre-education and posteducation mean VAS score between the VR group and the control group.

Before intervention, the mean±SD of knowledge in the intervention group and control groups were 11.34±3.9 and 10.82±3.6, respectively (*P*=0.514). After intervention, the knowledge score increased by 5.86±3.7points in the intervention group and by 2.63±3.3 points in the control group (Table 2, Fig. 3A). After intervention, the VR group (17.20±2.6) had significantly higher knowledge score than those of the control group (13.42±3.3, *P* <0.001) (Table 2, Fig. 3A).

**Table 2:** Comparison of scores of questionnaires for anxiety and knowledge between the VR group and the control group.

	VR group (*n*=44)	Control group (*n*=44)	*P*
Knowledge_Pre	11.34±3.9	10.82±3.6	0.514
Knowledge_Post	17.20±2.6	13.42±3.3	<0.001*
Knowledge_change	+5.86±3.7	+2.63±3.3	<0.001*
STAI-X-1_Pre	45.16±11.2	41.36±10.7	0.107
STAI-X-1_Post	41.02±11.2	40.52±11.5	0.837
STAI-X-1_change	−4.14±7.5	−0.84±5.7	0.023*
STAI-X-2_Pre	42.55±9.6	40.36±9.2	0.278
VAS_Pre	4.0±2.3	3.8±2.2	0.667
VAS_Post	3.5±1.9	3.6±1.8	0.725
VAS change	−0.5±1.2	−0.2±1.3	0.199
Satisfied score (40/44)	45.65±4.16		

Post, Postexplanation; Pre, Pre-explanation; STAI-X, State-Trait Anxiety Inventory-X; VAS, visual analog scale; VR, virtual reality.

*Statistically significant, *P*<0.05.

Regarding anxiety, STAI-X-1 scores between the intervention group and control group were similar at baseline. However, intervention group was decreased by −4.14±7.5 points, while by −0.84±5.7 in the control group (Table 2, Fig. 3B). After intervention, the average difference in the change in STAI-X1 score in the VR and control groups were −4.14±7.5 and −0.84±5.7 points, respectively, reflecting more reduced anxiety score in the intervention group. Regarding VAS score, no differences were observed for both before (4.0±2.3 vs. 3.8±2.2, *P*=0.667) and after the intervention (3.5±1.9 vs. 3.6±1.8, *P*=0.725). No difference was observed in change in VAS score of both groups (−0.5±1.2 vs. −0.2±1.3, *P*=0.199)

The satisfaction questionnaire was administered only in the VR group, and the satisfaction score (40 out of 44) was 45.65±4.16.

Table 3 shows the difference between patients who showed deceased STAI-X-1 score after the intervention (*n*=50) and those who did not (*n*=38). There were no significant differences in baseline characteristics such as age (59.9±6.8 vs. 57.6±8.2, *P*=0.162) sex (68.4 male vs. 80.0% male, *P*=0.214), educational background (52.6 above college vs. 52.0% above college, *P*=0.659), and previous psychiatric history (2.6 vs. 8.0%, *P*=0.384). There were no differences in pre- (10.92±3.9 vs. 11.20±3.6, *P*=0.730), post- (14.97±3.6 vs. 15.61±3.5, *P*=0.402), and change (+ 4.05±4.5 vs. + 4.43±3.4, *P*=0.657) in knowledge score between the two groups. There was significant difference in satisfaction score between the two groups among those who answered among the VR group (43.27±4.9 vs. 46.55±3.5, *P*=0.024). Proportion of VR education was significantly higher in the patients who showed decrease in STAI-X-1 score (36.8 vs. 60.0%, *P*=0.031).

**Table 3:** Comparison between patients who did not show decrease in STAI-X1 score and those with decrease in STAI-X1 score.

	STAI-X-1 not-decreased (*n*=38)	STAI-X-1 decreased (*n*=50)	*P*
Age (years)	59.9±6.8	57.6±8.2	0.162
Male	26 (68.4%)	40 (80.0%)	0.214
Educational background			0.659
Elementary school	2 (5.3%)	2 (4.0%)	
Middle school	5 (13.2%)	4 (8.0%)	
High school	11 (28.9%)	18 (36.0%)	
College	19 (50.0%)	24 (48.0%)	
Graduate school	1 (2.6%)	2 (4.0%)	
Previous psychiatric history	1 (2.6%)	4 (8.0%)	0.384
Previous treatment	2 (5.3%)	8 (16.0%)	0.116
Planned surgical extent			0.115
<10%	10 (26.3%)	3 (6.0%)	
15–20%	13 (34.2%)	21 (42.0%)	
33%	8 (21.1%)	12 (24.0%)	
50%	1 (2.6%)	3 (6.0%)	
70%	6 (15.8%)	11 (22.0%)	
Knowledge_Pre	10.92±3.9	11.20±3.6	0.730
Knowledge_Post	14.97±3.6	15.61±3.5	0.402
Knowledge_change	+4.05±4.5	+4.43±3.4	0.657
STAI-X-1_Pre	41.84±11.1	44.34±11.0	0.296
STAI-X-1_Post	45.00±11.2	37.56±10.3	0.002*
STAI-X-1_change	−3.16±3.2	+6.78±5.6	<0.001*
STAI-X-2_Pre	41.55±10.1	41.38±8.9	0.932
VAS_Pre	3.53±2.2	4.18±2.2	0.169
VAS_Post	3.68±2.0	3.44±1.7	0.533
VAS change	+0.16±1.3	−0.74±1.1	0.001*
Satisfaction score (40/44)	43.27±4.9 (n=11)	46.55±3.5 (n=29)	0.024*
VR education	14 (36.8%)	30 (60.0%)	0.031*

Post, Postexplanation; Pre, Pre-explanation; STAI-X, State-Trait Anxiety Inventory-X; VAS, visual analog scale; VR, virtual reality.

*Statistically significant, *P*<0.05.

Table 4 summarized the multivariable logistic regression model for potential factors that can be related to decrease in anxiety after intervention. Factors that showed significant relationship to decrease in STAI-X-1 score were planned surgical extent of ≥10% (HR=5.595, CI=1.418–22.073 *P*=0.014) and VR education (HR=2.571, CI=1.079–6.103, *P*=0.033). In the multivariable model, planned surgical extent of ≥10% (HR=11.529, CI=2.099–63.333, *P*=0.005) and VR education (HR=2.902, CI=1.097–7.674, *P*=0.032) were significantly related to decrease in STAI-X-1 score.

**Table 4:** Multivariable logistic regression analysis for factors related to decrease in STAI-X-1 score after intervention.

		Univariable			Multivariable		
	*n*	OR	CI	*P*	OR	CI	*P*
Sex (Male)	66	1.846	0.697–4.888	0.217			
Age ≥60 (years)	49	1.030	0.441–2.408	0.945			
Alcohol	7	1.014	0.213–4.829	0.986			
College education	46	0.975	0.419–2.269	0.953			
Previous psychiatric history	5	3.217	0.345–30.030	0.305			
Prior treatment of HCC	10	3.429	0.684–17.190	0.134	5.238	0.737–37.207	0.098
Planned surgical extent≥10%	75	5.595	1.418–22.073	0.014	11.529	2.099–63.333	0.005
VR intervention	44	2.571	1.079–6.103	0.033	2.902	1.097–7.674	0.032
Knowledge point ≥5 increase	39	1.714	0.725–4.056	0.220			

STAI-X, State-Trait Anxiety Inventory-X; VR, virtual reality.

## Discussion

This study investigated the impact of VR education on knowledge and anxiety among 88 patients diagnosed with HCC and scheduled for hepatic resection. The participants were evenly split into two groups: the VR group and the control group. While both groups had similar demographic and clinical characteristics, the VR group demonstrated a notable improvement in postexplanation knowledge scores compared to the control group, suggesting that VR education effectively enhanced patients’ understanding of their medical condition. Furthermore, the VR group showed a significant reduction in anxiety postintervention, as indicated by the STAI-X-1 scores. This finding was verified by multivariable analysis showing the statistically significant relationship to decrease in anxiety.

A further subgroup analysis, based on changes in STAI-X-1 scores postintervention, revealed two distinct groups: those whose anxiety levels remained unresolved or increased (STAI-X-1 not-decreased) and those who experienced a reduction in anxiety (STAI-X-1 decreased). The latter group, which benefited from a decrease in anxiety, showed a more pronounced response to the VR education, with a significant proportion (60.0%) receiving VR education and reporting higher satisfaction scores. In the multivariable analysis, VR intervention showed significant relationship for decreasing anxiety (HR=2.902, CI=1.097–7.674, *P*=0.032). Based on the finding that planned surgical extent ≥10% was highly related to decrease in anxiety (HR=11.529, CI=2.099–63.333, *P*=0.005), it can be interpreted that these patients can benefit more for education regarding anxiety. These findings underscore the potential of VR as a valuable tool in patient education, particularly in alleviating anxiety and enhancing knowledge among HCC patients before liver resection.

Recent research has explored the potential of VR in educating patients. One such investigation assessed the impact of VR-based training on immunotherapy knowledge among cancer patients undergoing immunotherapy^13^. Another study focused on patients set to undergo cardiac procedures, such as coronary artery bypass graft, surgical aortic valve replacement, and thoracic aortic aneurysm surgery^8^. This research highlighted that combining VR with 3D printed models for patient education not only elevated patient satisfaction but also effectively alleviated preoperative anxiety. Furthermore, a separate study indicated that VR educational videos, when offered to patients awaiting atrial fibrillation ablation, enhanced the quality of information provided, deepened procedural understanding, boosted patient satisfaction, and reduced procedural anxiety^14^. Wang *et al*.^5^ showed that VR-based education is an effective tool for improving patients’ knowledge and reducing their anxiety and depression levels during radiation therapy. Yang *et al*.^6^ also reported that patients undergoing arthroscopic knee surgery benefited from preoperative VR experiences through 3D reconstructive knee MRI, resulting in reduced surgery-related anxiety, higher overall satisfaction, and lowered postoperative stress levels.

VR-based education is being employed not just for patient instruction but also within the field of medical training^15^. A comprehensive 2D, 360-degree VR video was developed, illustrating an intracavitary brachytherapy procedure for treating cervical cancer. Trainees in radiation oncology were enlisted and divided into two distinct groups: the Integrated Headset VR (IHVR) and the Cardboard Viewer VR (CVVR). An evaluative survey gauged their confidence, understanding of the procedure, and their views on the VR technology’s efficacy, both presimulation and postsimulation. The findings indicated an enhancement in the trainees’ confidence and proficiency across both VR modalities. Both VR methods were perceived as engaging educational resources, offering immersive experience, and fostering active involvement. Notably, CVVR emerged as a cost-efficient educational medium, presenting a viable alternative to IHVR.

HCC is a tumor originating from the liver. Building a 3D model based on imaging is particularly suitable for these patients compared to those with other gastrointestinal malignancies. This is attributed to the liver’s characteristics as a solid, less mobile organ. In contrast, cancers of the stomach and colon are more difficult to model due to their mobility and structural complexity. Therefore, a VR platform can be effectively applied to patients with HCC, providing high-quality, intuitive 3D models.

Our study has some limitations. The study was conducted at a single institution with a relatively modest sample size of 88 patients, may have limited generalizability to broader populations or different clinical settings. This study excluded patients who were equal or older than 70-years of age. The reason for excluding the older aged group was to exclude the possibility of disturbance that the users might experience during VR technology. In general, old age can be a risk factor for experiencing motion sickness^16^. Since we designed this study to focus on those who can tolerate the VR experience, technically, our finding can only be limited to patients under 70-years of age. We did not collected data for side effects using the VR education. Nevertheless, any patient who is tolerable of using the device, we believe that this technology can be beneficial. This is further supported by the satisfaction analysis detailed in the Supplementary Table (Supplemental Digital Content 3, http://links.lww.com/JS9/C5). The responses indicated higher than moderate satisfaction, particularly for the question, ‘Did the VR education program make you feel at ease?’ No patient gave a score of 1 or 2, while the distribution of responses for scores 3, 4, and 5 was 6 (15%), 16 (40%), and 18 (45%) patients, respectively. The reliance on self-reported measures, such as the STAI-X and VAS scores, introduces potential subjectivity in assessing anxiety, which could be influenced by various external factors not controlled for in the study. Additionally, the effectiveness of the VR education might be influenced by participants’ familiarity with technology, and the novelty of the VR experience could introduce a placebo effect, potentially skewing the perceived benefits. The limitation in using less validated questionnaires can be a limitation while organizing a knowledge test questionnaire just for the study was a novel approach. The knowledge that is required for certain patients differ from other patients with different conditions. Therefore, it is difficult to find and already-validated questionnaire to evaluate the knowledge that is relevant for the patient.

Whether VR is better than education program using tablets cannot be determined by this single study. Since 3-dimensional model and educational videos can also be presented using those devices, the experience that the users may have inside the VR itself should be judged between those devices if we plan to analyze the impact of VR platform itself. Technically, there may be benefit of using a VR device compared to using tablet, since VR can provide immersive experiences. Users can dive into the platform and other distraction from the environment can be prevented.

Another point that must be mentioned is the feasibility of the program itself. Using the program for multiuser-access requires wireless connection through the router between the devices. The technical hurdle exists for applying this kind of program since patient-specific 3D model is required for visualizing the liver.

Nevertheless, our study utilized a rigorous randomized controlled trial design to innovatively explore the benefits of VR technology in patient education, assessing both anxiety and knowledge. The inclusion of a detailed subgroup analysis further enriched our insights, highlighting the nuanced impact of the intervention on different patient populations.

## Conclusion

In this randomized controlled trial, VR education demonstrated a promising potential in enhancing the understanding and reducing the anxiety of patients diagnosed with HCC before liver resection. The significant improvements in postintervention knowledge scores and the notable reduction in anxiety among a subset of patients underscore the value of VR as an innovative and effective tool in patient education. In clinical areas that are difficult for patients to understand, incorporating immersive technologies such as VR can provide a more personalized and impactful patient experience, improving patient understanding and reducing anxiety.

## Ethical approval

The study was approved by the Institutional Review Board (IRB) of Samsung Medical Center (IRB No. 2021-11-017-007), and all study participants provided written informed consent.

## Consent

Written informed consent was obtained from the patient for publication of this study. A copy of the written consent is available for review by the Editor-in-Chief of this journal on request.

## Sources of funding

This study was supported by Samsung Medical Center Grant #SMO1220681, and the Korea Health Technology R&D project through the Korea Health Industry Development Institute (KHIDI), funded by the Ministry of Health & Welfare (HI23C038700).

## Author contribution

J.Y.: study design, manuscript draft, and revision of manuscript; S.L.: study design, 3D modeling, and education video design; J.R.: study design, manuscript draft, and revision of manuscript; J.C., G.-S.C., J.M.K., J.-W.J., and D.K.: study design; H.L.: study design, program design for the study, and VR platform.

## Conflicts of interest disclosure

Heesuk Lee is the C.E.O of VRAD of which the VR platform for the study was designed.

## Research registration unique identifying number (UIN)

The study protocol was registered at CRIS.nih.go.kr (registration number: KCT0006924) before the start of participant enrollment.

## Guarantor

Jinsoo Rhu the corresponding author.

## Data availability statement

Data can be provided by researcher who request for it under the permission of the institution.

## Provenance and peer review

This study was not invited for this.

## Supplementary Material
