# Supplementary material for: Impact of virtual reality education on disease-specific knowledge and anxiety for hepatocellular carcinoma patient scheduled for liver resection: a randomized controlled study
Source: Int J Surg. 2024 Feb 21;110(5):2810–7. doi: 10.1097/JS9.0000000000001197 (PMC11093422; doi:10.1097/JS9.0000000000001197)
Supplement: Supplementary file 3 [file js9-110-2810-s003.doc]

**Questionnaires for Knowledge Assessment**

1. Where is the liver located in the body? (1 point)


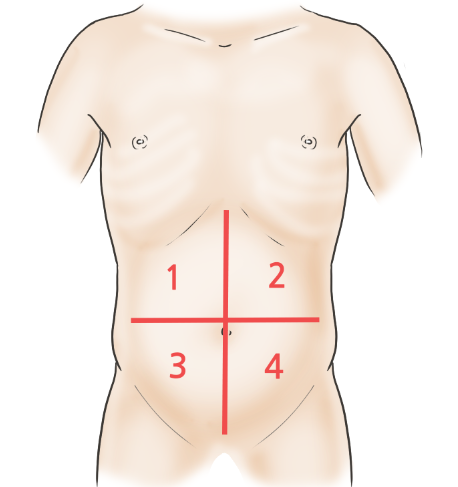


2. After the liver resection, the remaining liver regenerates back to its original shape. (True / False) (1 point)

3. After liver resection, how long does it take for the liver to regenerate back? (2 points)

① One week

② 3 months

③ 6 months

④ 12 months

⑤ It doesn't regenerate back.

4. Which organ is most likely to be removed along with the liver during liver resection? (1 point)

① Stomach

② Pancreas

③ Gallbladder

④ Spleen

⑤ Colon

5. Removing the gallbladder does not cause any significant disruption to daily life. (True / False) (1 point)

6. What is the purpose of your liver resection? (2 points)

① Removal of hepatocellular carcinoma

② Removal of intrahepatic cholangiocarcinoma

③ Removal of hepatic cystic tumor

④ Removal of hepatic hemangioma

⑤ Removal of hepatic simple cyst

7. Choose the image that best describes the resection boundary during liver resection. (1 point)

(Note: Yellow mass is hepatocellular carcinoma, Red area is liver, Blue line is resection boundary)

①
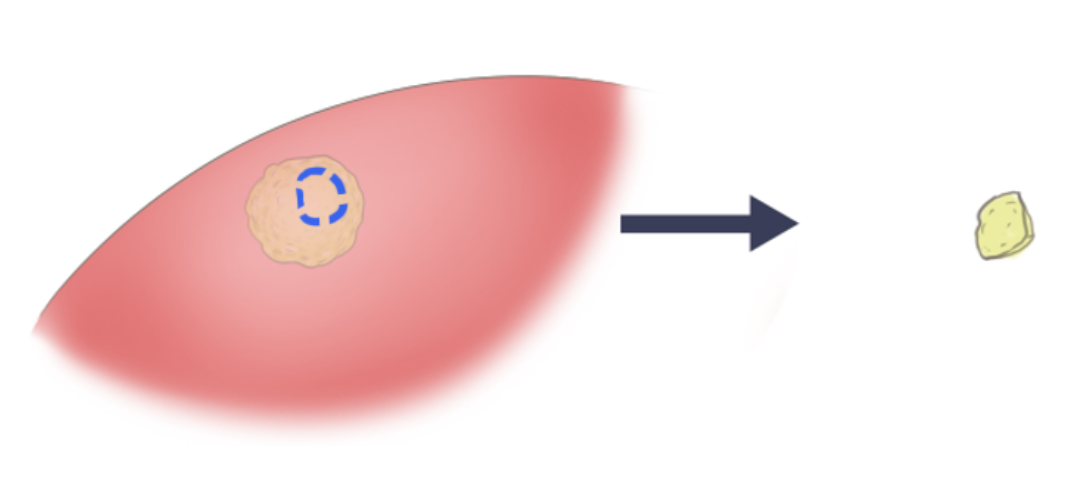


②
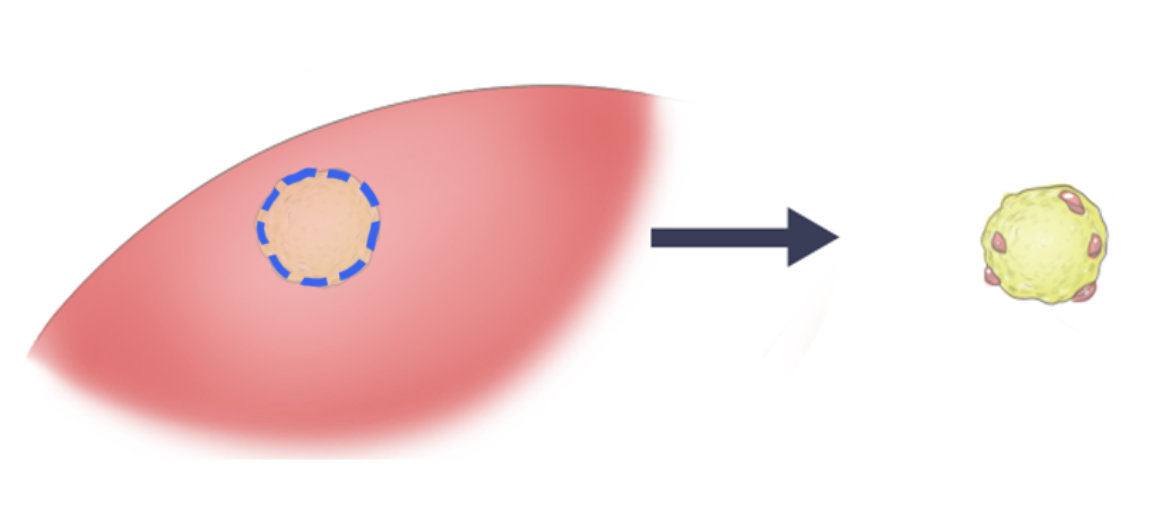


③


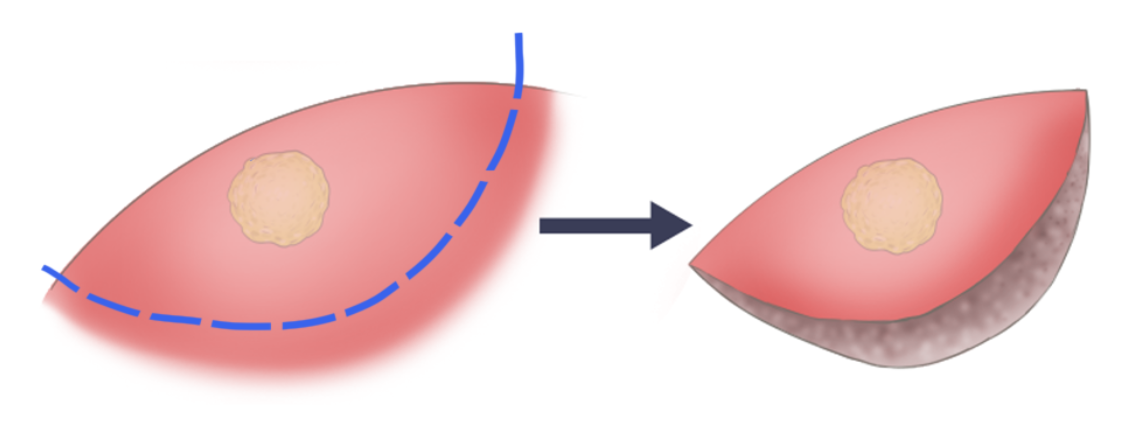


8. Mark all segment(s) where your hepatocellular carcinoma is located, which is planned for liver resection. (3 points)


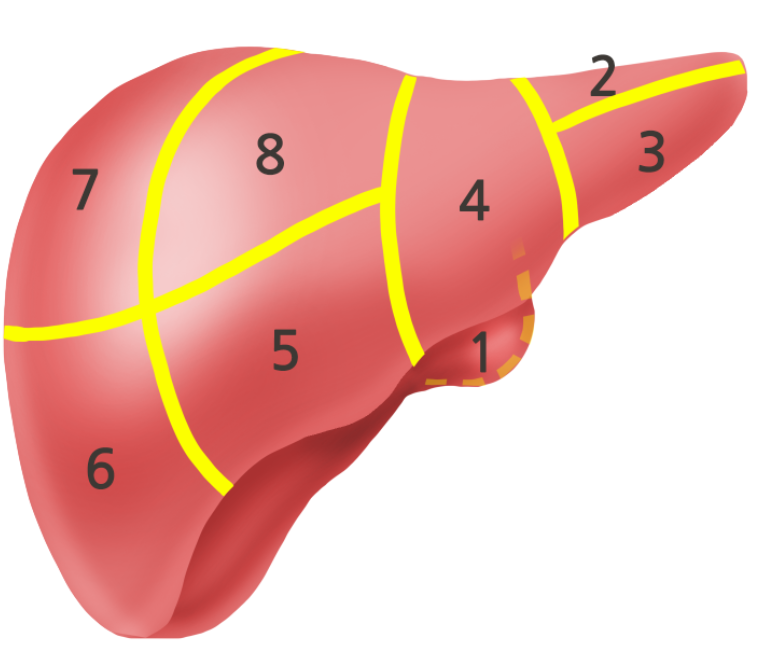


9. Mark all segment(s) of planned liver resection you will undergo. (3 points)
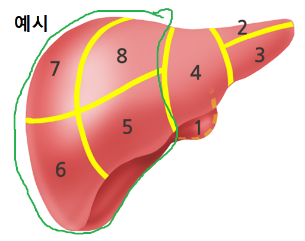
.


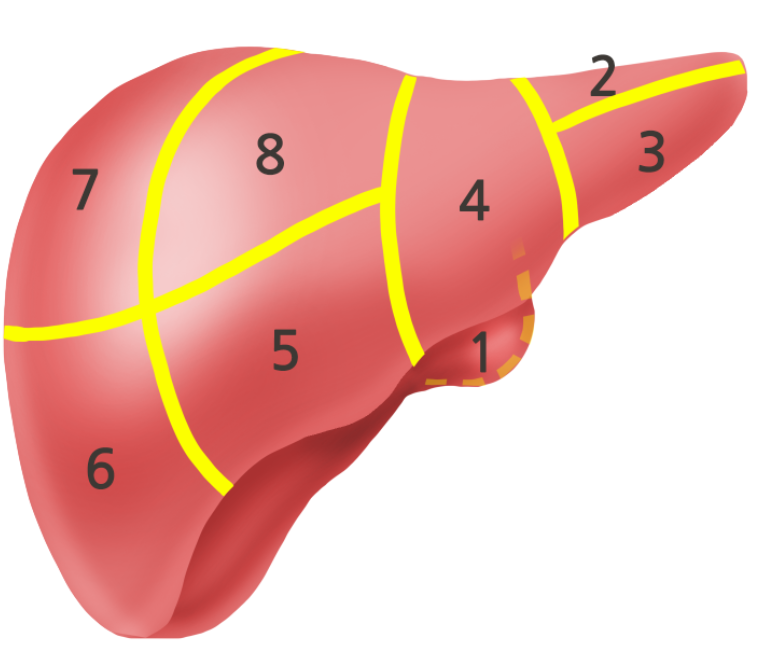


10. Which method of liver resection is planned for your operation? (1 point)

① Open surgery

② Laparoscopic surgery

③ Robotic surgery

④ Endoscopic surgery

⑤ Radiofrequency ablation

⑥ Chemoembolization

11. What percentage of the total liver size is the planned liver resection? (2 points)

① Less than 10%


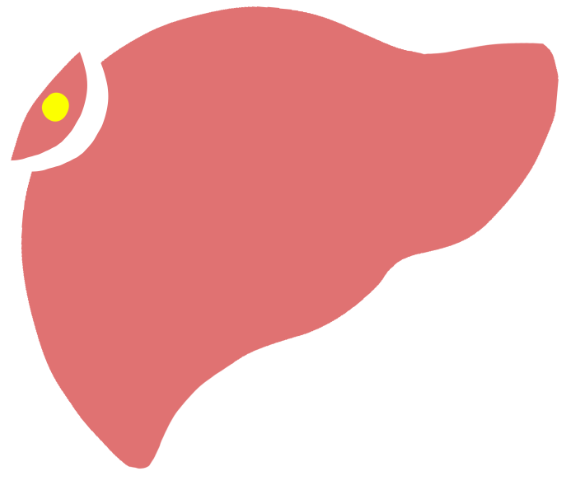


② Around 20%


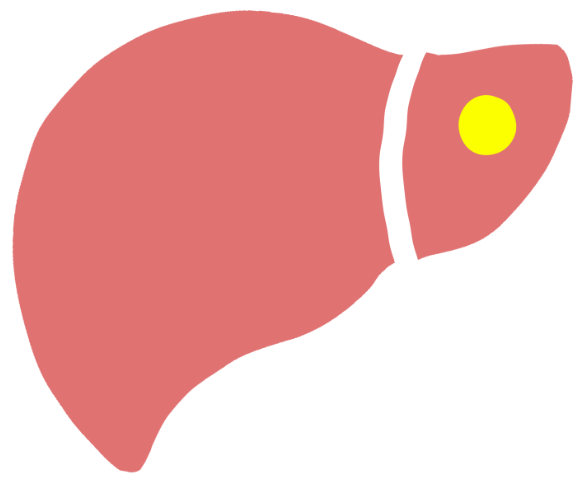

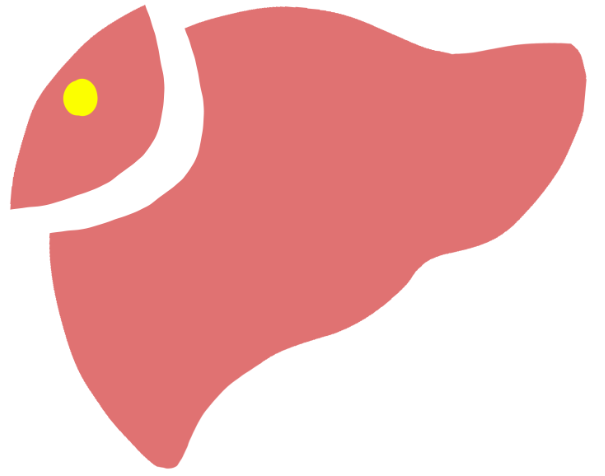


③ Around 30-40%


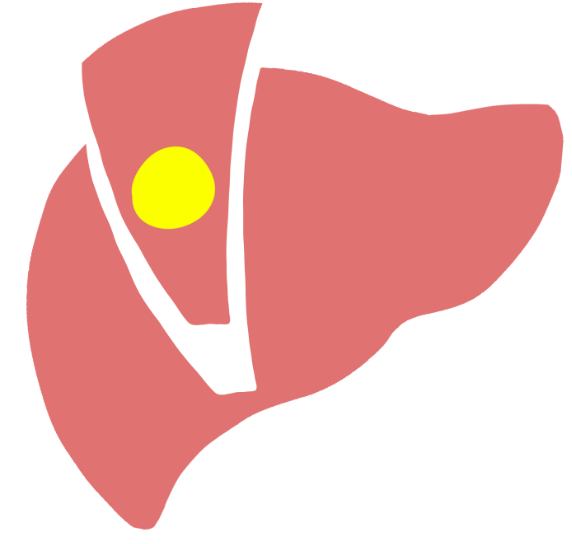

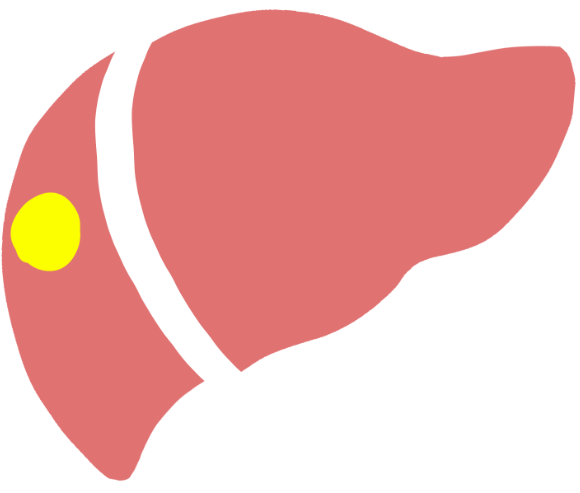

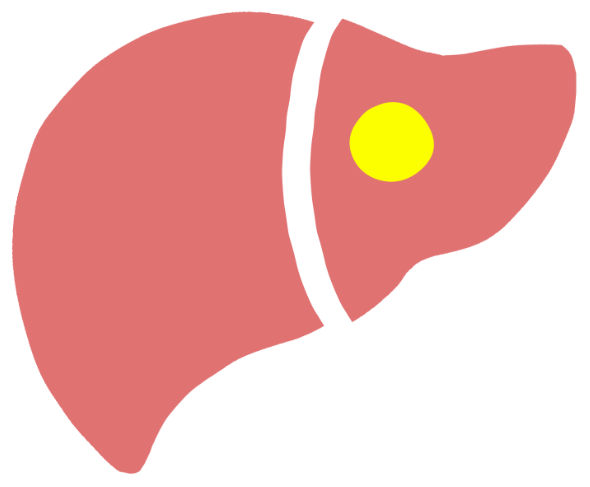


④ 50-70%


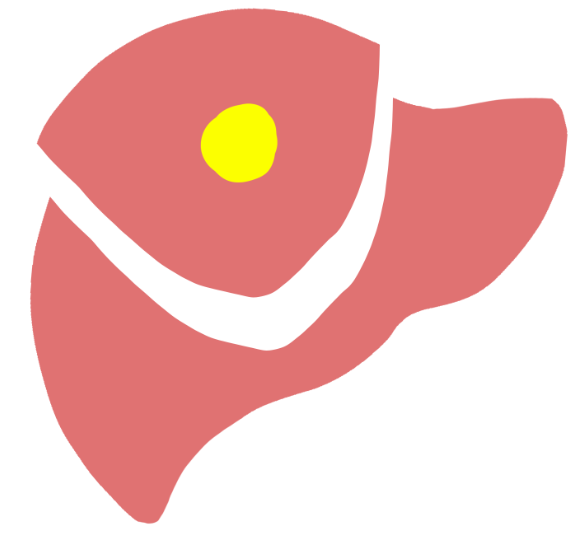

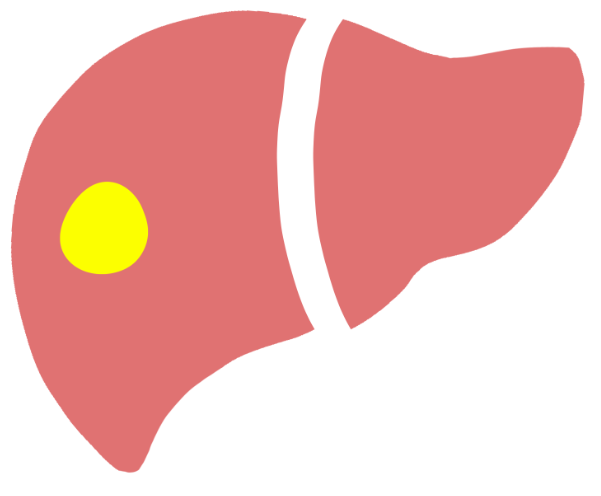


12. Which of the following is a scar from laparoscopic liver resection? (1 point)

① ②


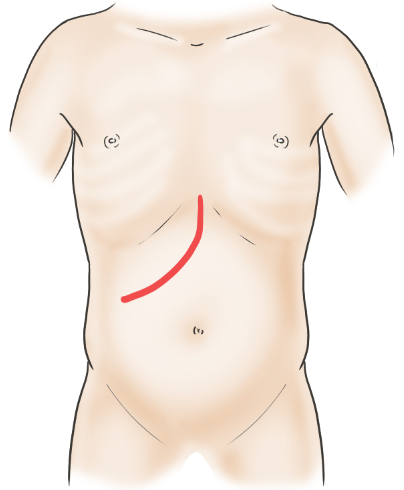

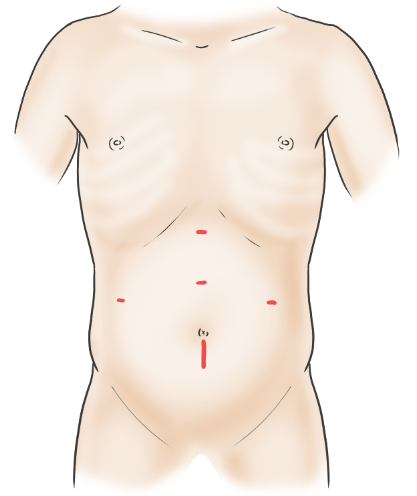


13. What is the average hospital stay after liver resection? (1 point)

① 1-3 days after surgery

② 5-7 days after surgery

③ 10 days after surgery

④ 15 days after surgery

|  | Questions | Not at all | | | Strongly so | | |
| --- | --- | --- | --- | --- | --- | --- | --- |
|  |  | 1← →5 | | | | | |
| 1 | The VR education program helped me understand my disease. | 1 | 2 | 3 | | 4 | 5 |
| 2 | The VR education program made me feel at ease. | 1 | 2 | 3 | | 4 | 5 |
| 3 | The VR education program made me more aware of my condition. | 1 | 2 | 3 | | 4 | 5 |
| 4 | The VR education program helped me trust the medical staff. | 1 | 2 | 3 | | 4 | 5 |
| 5 | Patients facing surgery should undergo the VR education program. | 1 | 2 | 3 | | 4 | 5 |
| 6 | Before experiencing the VR education program, I was unaware of my condition. | 1 | 2 | 3 | | 4 | 5 |
| 7 | The VR education program was easy to understand. | 1 | 2 | 3 | | 4 | 5 |
| 8 | The VR education program gave me the courage to undergo surgery. | 1 | 2 | 3 | | 4 | 5 |
| 9 | The National Health Insurance Service should provide enough support for the VR education program. | 1 | 2 | 3 | | 4 | 5 |
| 10 | I am generally satisfied with the VR education program. | 1 | 2 | 3 | | 4 | 5 |
| 11 | If you were to pay for the VR education program, how much are you willing to pay? | won(\) | | | | | |

Gender: Male/Female Age: Date: / /

Satisfaction analysis

|  |  | 1 | 2 | 3 | 4 | 5 |
| --- | --- | --- | --- | --- | --- | --- |
| 1 | The VR education program helped me understand my disease. | - | - | - | 6  (15%) | 34  (85%) |
| 2 | The VR education program made me feel at ease. | - | - | 6  (15%) | 16  (40%) | 18  (45%) |
| 3 | The VR education program made me more aware of my condition. | - | - | 1  (2.5%) | 10  (25%) | 29  (72.5%) |
| 4 | The VR education program helped me trust the medical staff. | - | - | - | 9  (22.5%) | 31  (77.5%) |
| 5 | Patients facing surgery should undergo the VR education program. | - | - | 1  (2.5%) | 14  (35.0%) | 25  (62.5%) |
| 6 | Before experiencing the VR education program, I was unaware of my condition. | 2  (5%) | 2  (5%) | 13  (32.5%) | 6  (15%) | 17  (42.5%) |
| 7 | The VR education program was easy to understand. | - | - | 1  (2.5%) | 6  (15.0%) | 33  (82.5%) |
| 8 | The VR education program gave me the courage to undergo surgery. | - | 1  (2.5%) | 4  (10%) | 12  (30.0%) | 23  (57.5%) |
| 9 | The National Health Insurance Service should provide enough support for the VR education program. | - | - | 2  (5.0%) | 9 (22.5%) | 29 (72.5%) |
| 10 | I am generally satisfied with the VR education program. | - | - | 1  (2.5%) | 5  (12.5%) | 34  (85.0%) |
| 11 | If you were to pay for the VR education program, how much are you willing to pay? | won(\) | | | | |
